# *Siegesbeckia Glabrescens* Extract Ameliorates Immobilization‐Induced Muscle Atrophy by Regulating the Akt/mTOR/FoxO3a Signaling Pathways in Mice

**DOI:** 10.1002/fsn3.71005

**Published:** 2025-09-26

**Authors:** Sungmin Han, Jae‐Kwan Hwang, Mi‐Bo Kim

**Affiliations:** ^1^ Graduate School of Bioindustrial Engineering Yonsei University Seoul Republic of Korea; ^2^ Department of Food Science and Nutrition Pukyong National University Busan Republic of Korea

**Keywords:** Akt/mTOR/FoxO3a signaling pathway, immobilization, muscle atrophy, *Siegesbeckia glabrescens* hot water extract

## Abstract

*Siegesbeckia glabrescens*, traditionally used for its anti‐inflammatory properties and potential musculoskeletal benefits. In the present study, we investigated whether a standardized hot water extract of *S. glabrescens* (SGE), rich in the bioactive compound kirenol, could mitigate muscle atrophy caused by immobilization in a mouse model. Male C57BL/6J mice underwent hindlimb immobilization for 1 week to induce muscle atrophy, followed by 1 week of daily oral treatments with either saline or SGE (150 or 300 mg/kg/day). SGE significantly prevented the decline in muscle mass (5.1%–22.7%, *p* < 0.05), strength (26.0%–36.6%, *p* < 0.05), volume, and muscle fiber cross‐sectional area (21.6%–29.8%, *p* < 0.05) caused by immobilization. In the tibialis anterior muscle, SGE mitigated immobilization‐induced elevations in protein degradation markers, including muscle RING‐finger protein‐1 and muscle atrophy F‐box, by inhibiting the nuclear translocation of forkhead box O3a. Also, SGE countered reductions in protein synthesis indicators, including mammalian target of rapamycin, 70‐kDa ribosomal protein S6 kinase, and 4E binding protein 1, through activation of the phosphatidylinositol 3‐kinase/Akt pathway. Furthermore, kirenol attenuated tumor necrosis factor‐alpha‐driven muscle atrophy in L6 myotubes by modulating both protein synthesis and degradation processes. Collectively, these findings suggest that SGE containing kirenol maintains the equilibrium between protein degradation and protein synthesis, thereby ameliorating immobilization‐induced muscle atrophy.

## Graphical abstract

*Siegesbeckia glabrescens* extract enriched with kirenol alleviates skeletal muscle atrophy by enhancing protein synthesis and decreasing protein degradation via the Akt/mTOR/FoxO3a signaling pathway.

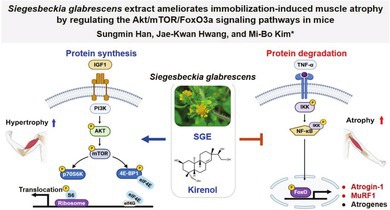

## 1 Introduction

Skeletal muscle atrophy, marked by a gradual loss of muscle mass and strength, can develop in a range of immobilizing situations such as spinal cord injury, extended bed rest, microgravity exposure, and prolonged intensive care (Ji and Yeo 2019; Zarzhevsky et al. 2001). This condition is marked by reductions in both the size and number of muscle fibers, along with an imbalance between protein synthesis and degradation, which can result in reduced physical capabilities and a diminished quality of life (Barreiro 2018). In the pathogenesis of immobilization, chronic inflammation and oxidative stress play pivotal roles that disturb the equilibrium between protein synthesis and breakdown (Phillips and McGlory 2014; Powers et al. 2011). High levels of reactive oxygen species (ROS) generation and inflammatory cytokines impede protein synthesis by suppressing the mammalian target of rapamycin (mTOR) pathway, while simultaneously accelerating protein breakdown via the ubiquitin‐proteasome system (Phillips and McGlory 2014; Powers et al. 2011). Therefore, it is essential to identify natural bioactive ingredients that possess antioxidant and anti‐inflammatory properties, as they could be valuable resources for mitigating skeletal muscle mass and functional losses associated with muscle atrophy.

*Siegesbeckia* Herba, a traditional herbal medicine, officially refers to the edible aerial parts of three herbs: *Siegesbeckia orientalis* L., *Siegesbeckia glabrescens* Makino, and *Siegesbeckia pubescens* Makino. These plants are widely distributed across various regions, including China, Republic of Korea, Japan, and Vietnam (Tao et al. 2021). *Siegesbeckia* Herba, commonly referred to as “Xi‐Xian Cao” has been acknowledged as a significant traditional Chinese medicine since the Tang dynasty (Pradhan et al. 2018). Traditionally, this medicinal herb has been used to manage various inflammatory conditions, including musculoskeletal weakness, quadriplegia, hemiplegia, lower back and knee pain, rubella wet sores, and rheumatism (Wang et al. 2021). Also, studies have demonstrated that *Siegesbeckia* Herba has anti‐inflammatory (Linghu et al. 2020), antioxidant (Shim et al. 2021), antiadipogenic (Kim, Kim, and Hwang 2020), antibacterial (Yang et al. 2016), antiphotoaging (Kim et al. 2017), and antithrombotic properties (Lv et al. 2023). Furthermore, kirenol, a main active diterpenoid component in *Siegesbeckia* Herba, possesses anti‐inflammatory (Nasir et al. 2022), antiadipogenic (Kim, Song, Hwang, et al. 2014), antiarthritic (Lu et al. 2012), and osteoblast differentiation‐enhancing properties (Kim, Song, Kim, et al. 2014). In our previous research, an *S. orientalis* extract standardized to its kirenol content was shown to enhance exercise endurance by stimulating mitochondrial biogenesis in the skeletal muscles of obese mice and L6 myotubes, which suggests that it has a beneficial effect on exercise capacity by promoting mitochondrial biogenesis in skeletal muscle (Kim et al. 2019). In particular, *S. glabrescens* was known to possess a distinct chemical profile, notably exhibiting higher levels of flavonoids and phenolic acids compared to *S. orientalis* (Gao et al. 2018). Also, *S. glabrescens* has been reported to display superior anti‐inflammatory and antioxidant activities in both in vitro and in vivo models (Gao et al. 2018; Kim et al. 2017; Shim et al. 2021). These evidences suggest that *S. glabrescens* may have greater therapeutic potential for preventing skeletal muscle atrophy compared to other *Siegesbeckia* species. Nevertheless, whether *S. glabrescens* can protect against skeletal muscle atrophy remains to be determined. Hence, in this study, we examined whether a standardized hot water extract of *S. glabrescens* (SGE), containing kirenol, can alleviate immobilization‐induced muscle atrophy in C57BL/6J mice to elucidate its potential role in skeletal muscle deterioration. We hypothesized that SGE mitigates skeletal muscle atrophy by regulating the protein kinase B (Akt)/mTOR/forkhead box O3a (FoxO3a) pathways to enhance protein synthesis and suppress degradation, thereby restoring the balance between these processes in the immobilized muscles of C57BL/6J mice.

## 2 Materials and Methods

### 2.1 Preparation of SGE

**FIGURE 1:**
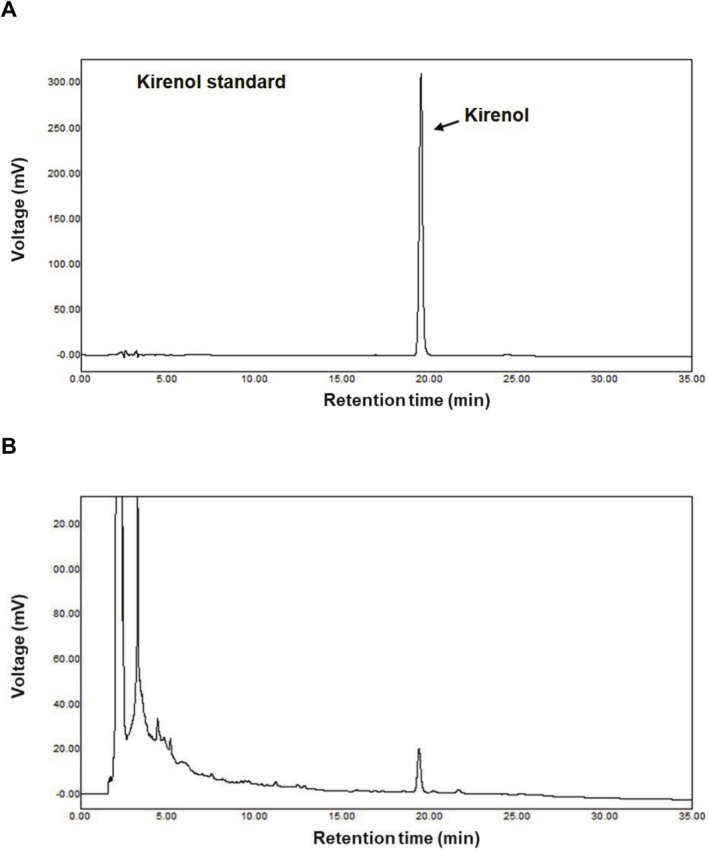
HPLC chromatograms. (A) HPLC chromatograms of kirenol standard and (B) SGE.

Dried aerial parts of *S. glabrescens* were purchased from Biocare (Gyeonggi‐do, Republic of Korea) and taxonomically identified by Prof. Wonku Kang at the College of Pharmacy, Chung‐Ang University, Seoul, Republic of Korea. A voucher specimen (YBT‐SG2015) has been deposited at the Department of Biotechnology, Yonsei University. Dried and ground *S*. *glabrescens* (100 g) was extracted with hot water at 95°C for 3 h, as per a previously published method (Kim et al.  2017). The extract was filtrated using filter paper and concentrated using the rotary vacuum evaporator (Laborota 4000 efficient, Heidolph Instruments GmbH and Co. KG., Schwabach, Germany) to obtain the SGE with a yield of 19.4% (w/w). The SGE was standardized for the content of kirenol using high‐performance liquid chromatography analysis, as per a previously published method (Kim, Park, et al. 2014). The amount of kirenol in SGE was determined to be 0.67% (w/w) based on a standard curve using pure kirenol (Institute for Korea Traditional Medical Industry in Daegu, Republic of Korea) (Figure 1).

### 2.2 Animal Studies

Seven‐week‐old male C57BL/6J mice were obtained from Young Bio (Seongnam, Republic of Korea) and maintained under a 12‐h light/dark cycle at 25°C ± 2°C with 55% ± 5% relative humidity. They had free access to standard chow and water. After 1 week of acclimatization, the mice were randomly assigned to four groups (*n* = 8 per group): (1) a nonimmobilized control (CON), (2) an immobilization‐only group (IM), (3) an immobilized group treated with SGE at 150 mg/kg/day (IM + SGE150), and (4) an immobilized group treated with SGE at 300 mg/kg/day (IM + SGE300). Sample size was determined based on our previous study investigating skeletal muscle atrophy and pharmacological interventions in mice (Hwang et al. 2024; C. Kim, M.‐B. Kim, and J.‐K. Hwang, 2020; Yoo et al. 2024), which typically used six to eight mice per group to achieve statistically meaningful results. Muscle atrophy was induced by immobilizing the right hindlimb for 1 week, while the left hindlimb served as a nonimmobilized control, as previously described (Kim, Kim, and Hwang 2020). Under anesthesia with 350 mg/kg of 2,2,2‐tribromoethanol (Sigma‐Aldrich, St. Louis, MO, USA), a surgical staple was inserted from the foot into the distal gastrocnemius (GA) of the right hindlimb using a skin stapler (F‐35 W; Unidus, Seoul, Republic of Korea). After the 1‐week immobilization period, the staple was removed while the mice remained anesthetized. On that same day, the IM + SGE150 and IM + SGE300 groups received oral SGE daily for 1 week, while the CON and IM groups were administered saline. Body weight and food intake were measured twice weekly. At the end of the treatment period, grip strength and muscle volumes were assessed before the mice were euthanized via cardiac puncture under anesthesia. Liver and spleen weights were measured at the end of the experiment to assess potential systemic toxicity of SGE treatment. No significant differences in liver and spleen weights were observed between the SGE‐treated and control groups (Figure S1). The GA, soleus (SOL), tibialis anterior (TA), and extensor digitorum longus (EDL) muscles were rapidly harvested and either snap‐frozen in liquid nitrogen for gene expression analyses or fixed in 10% formalin for histological examinations. All animal experiments were conducted in accordance with the guidelines of the Yonsei University Institutional Animal Care and Use Committee (IACUC) and were approved under protocol number IACUC‐A‐201608‐377‐04.

### 2.3 Grip Strength Test

A grip strength test was performed to assess the forelimb and hindlimb strength of mice using a Chatillon force measurement device (Columbus Instrument, Columbus, OH, USA), following previously published procedures (Kim, Kim, and Hwang 2020).

### 2.4 Micro‐Computed Tomography (Micro‐CT)

Muscle volume was assessed via micro‐CT imaging using a Siemens Inveon system for PET/CT/SPECT (Knoxville, TN, USA), located at the Center for Evaluation of Biomaterials (Pohang Technopark, Pohang, Republic of Korea).

### 2.5 Histological Analysis of TA

TA muscle samples preserved in 10% formalin were stained with hematoxylin and eosin (H&E). The stained sections were examined under a CK40 inverted microscope (Olympus, Tokyo, Japan) equipped with a T500 camera (eXcope, Daejeon, Republic of Korea). The cross‐sectional area (CSA) of individual muscle fibers was quantified using ImageJ software (National Institutes of Health, Bethesda, MD, USA). Measurements were taken from one or two fields per mouse.

### 2.6 Reverse Transcription–Polymerase Chain Reaction (RT‐PCR)

Total RNA was extracted from TA muscle tissues and L6 myoblasts using TRIzol reagent (Takara Bio, Otsu, Japan). cDNA was synthesized and amplified with the GeneAmp PCR System 2700 (Applied Biosystems, Foster City, CA, USA), employing Reverse Transcriptase Premix and PCR Premix (ELPIS‐Biotech, Daejeon, Korea), following previously published protocols (Kim, Kim, and Hwang 2020). The resulting PCR products were resolved on a 1.5% agarose gel, stained using Loading STAR Dye (Dyne Bio, Seongnam, Republic of Korea), and visualized with a G:BOX EF imaging system (Syngene, Cambridge, UK). The specific primer sequences are listed in Table 1.

**TABLE 1:** Primer sequences used in reverse transcription–polymerase chain reaction.

Origin	Gene	Direction	Sequence (5′–3′)
Mouse	MuRF1	Forward	CCCTCTGCCCACCATTTACA
		Reverse	TTCTGTCTGCGGCTGTTGTC
	Atrogin‐1	Forward	CAAGAAGAGAGCAGTATGGGGTC
		Reverse	TTGAGGGGAAAGTGAGACGGA
	β‐Actin	Forward	GCAGGAGTACGATGAGTCCG
		Reverse	ACGCAGCTCAGTAACAGTCC

### 2.7 Western Blot Analysis

Western blot analysis was performed to explore the molecular pathways linked to protein breakdown and synthesis, as previously detailed (Kim, Kim, and Hwang 2020). Primary antibodies, diluted 1:1000, included phospho (p)‐FoxO3a, FoxO3a, p‐mTOR, mTOR, p‐70‐kDa ribosomal protein S6 kinase (p70S6K), p70S6K, p‐4E binding protein 1 (p‐4EBP1), 4EBP1, p‐phosphatidylinositol 3‐kinase (p‐PI3K), PI3K, p‐Akt, Akt, and α‐Tubulin (all from Cell Signaling, Beverly, MA, USA). Protein detection and quantification were performed with the G:BOX EF imaging system and the GeneSys software (Syngene).

### 2.8 Cell Culture

Rat myoblasts L6 cell line (CRL‐1458, American Type Culture Collection, Manassas, VA, USA) were maintained in Dulbecco's Modified Eagle's Medium (DMEM; Hyclone, Logan, UT, USA) containing 10% fetal bovine serum (FBS; Hyclone) and antibiotics (100 U/mL penicillin, 100 μg/mL streptomycin). Cells were grown at 37°C in a 5% CO_2_ environment until they reached 70%–80% confluence. Myotube differentiation was induced by replacing the growth medium with DMEM containing 2% horse serum (Gibco, Gaithersburg, MD, USA) for 6 days, with medium changes every 2 days. After differentiation, the cells were simultaneously treated with 6 ng/mL tumor necrosis factor‐alpha (TNF‐α) (PeproTech, Rocky Hill, NJ, USA) and kirenol at 10, 20, or 40 μM for 24 h. A concentration of 6 ng/mL TNF‐α was selected to induce muscle atrophy, as serum levels of 1–6 ng/mL observed in patients with inflammatory diseases are sufficient to promote muscle protein loss without significant cytotoxicity (Li and Reid 2000). Kirenol concentrations were determined based on reported levels in *Siegesbeckiae* Herba (4–49 μmol/g dry weight) and supported by cytotoxicity assays (Wang et al. 2021).

### 2.9 Cell Viability

Cell viability was assessed using the colorimetric thiazolyl blue tetrazolium bromide (MTT) assay. L6 myoblasts were seeded and treated with various concentrations (10–60 μM) of kirenol for 24 h. Following treatment, MTT solution (0.5 mg/mL) was added to the cells, and the cells were incubated at 37°C for 3 h. After the incubation, the supernatant was removed, and the resulting formazan crystals were dissolved in dimethyl sulfoxide. The absorbance was determined at 540 nm using a VERSAmax tunable microplate reader (Molecular Devices Inc., Sunnyvale, CA, USA).

### 2.10 Statistical Analysis

Group differences were assessed using one‐way ANOVA, followed by Tukey's post hoc test, or via unpaired *t*‐tests when necessary. Statistical calculations were performed with GraphPad Prism 10.0 (GraphPad Software, La Jolla, CA, USA). Differences were considered significant when *p* < 0.05, and data are expressed as mean ± standard error of the mean (SEM).

## 3 Results

### 3.1 SGE Prevented Muscle Mass Loss in Mice With Immobilization‐Induced Muscle Atrophy

**FIGURE 2:**
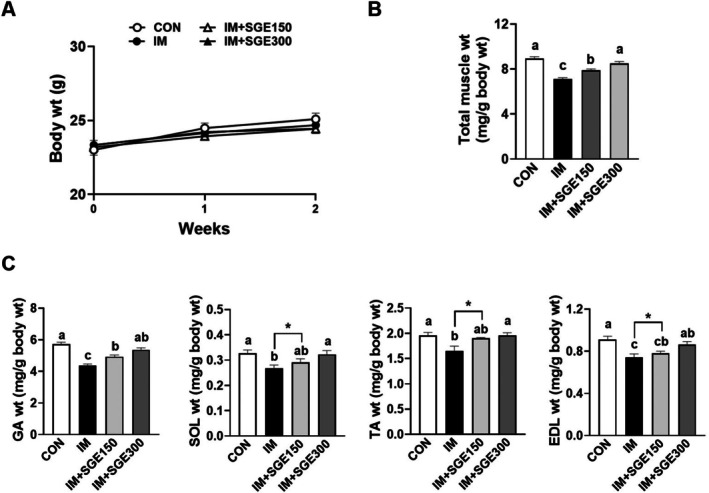
Protective effects of SGE on muscle mass in mice with immobilization‐induced muscle atrophy. Mice underwent 1 week of hindlimb immobilization to induce skeletal muscle atrophy, followed by 1 week of oral saline or SGE (150 or 300 mg/kg/day). (A) Body weight. (B) Total muscle weight. (C) Individual muscle weights of GA, SOL, TA, and EDL. Data are shown as mean ± SEM. *n* = 8 per group. Bars sharing the same letter do not differ significantly (*p* < 0.05) based on one‐way ANOVA with Tukey's post hoc test or an unpaired *t*‐test. *p* values for the unpaired *t*‐test are indicated.

Since immobilization‐related muscle atrophy is typified by a decrease in muscle mass, we first measured the weights of four different muscle types in immobilized mice. Throughout the experiment, there were no notable differences in body weight across any of the groups (Figure 2A). In the IM group, total muscle weight was significantly reduced by 20% compared with the CON group. However, administration of SGE significantly alleviated the loss in total muscle weight (Cohen's d: 2.0, 3.6 for IM vs. IM + SGE150 or IM + SGE300; Figure 2B). Although GA (Cohen's d: −1.73, −3.07 for IM vs. IM + SGE150 or IM + SGE300), SOL (Cohen's d: −0.56, −1.37 for IM vs. IM + SGE150 or IM + SGE300), TA (Cohen's d: −1.14, −1.71 for IM vs. IM + SGE150 or IM + SGE300), and EDL (Cohen's d: −0.47, 1.52 for IM vs. IM + SGE150 or IM + SGE300) muscles each declined by over 15% in the IM group compared with the CON group; SGE mitigated these decreases in a dose‐dependent manner in all tested muscle types (Figure 2C).

### 3.2 SGE Alleviated the Reductions in Grip Strength, Muscle Volume, and Muscle Fiber CSA Associated With Immobilization‐Induced Muscle Atrophy

**FIGURE 3:**
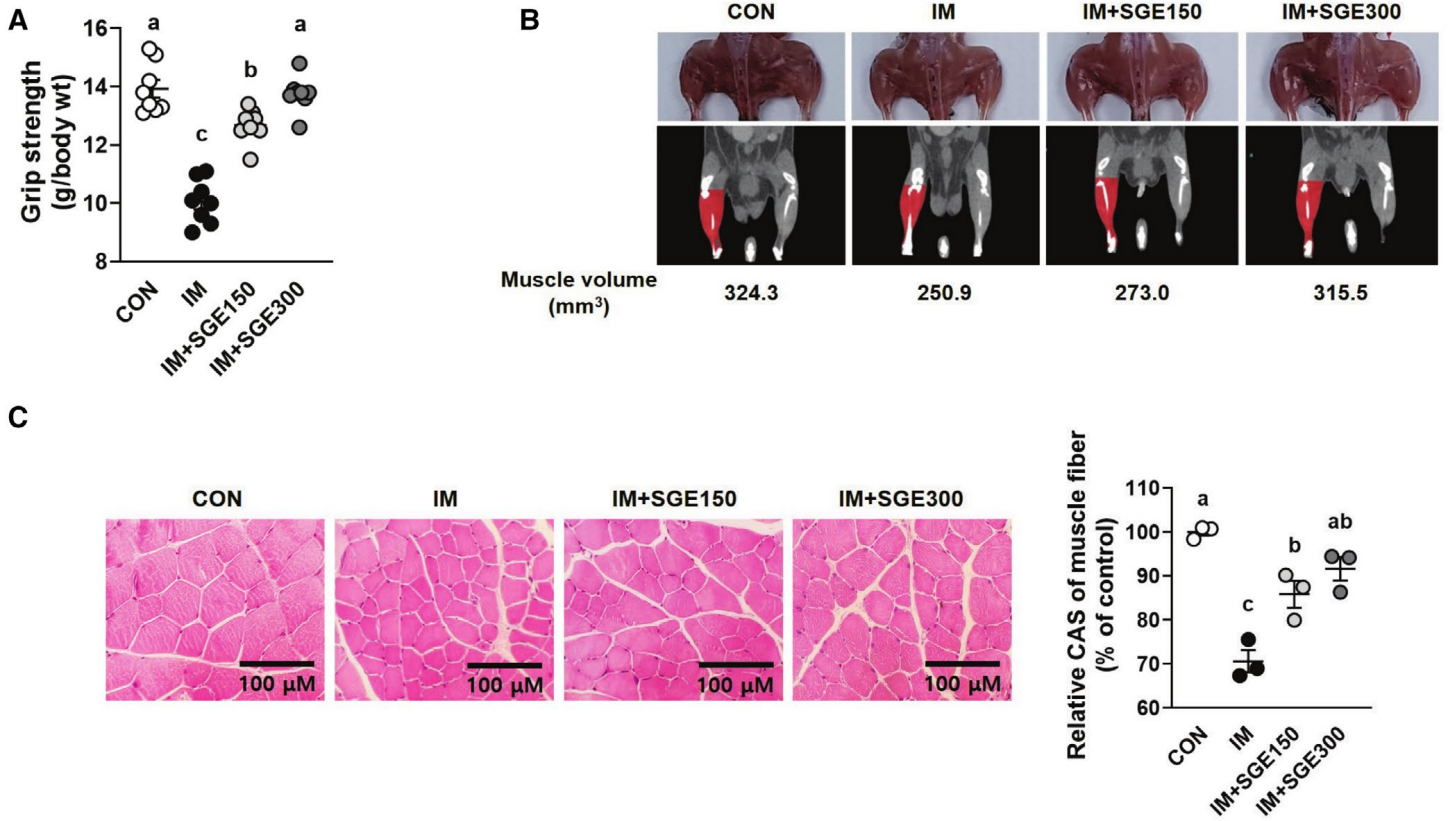
Effects of SGE on grip strength, muscle volume, and CSA myofibers in immobilization‐induced muscle atrophy. Mice underwent 1 week of hindlimb immobilization to induce skeletal muscle atrophy, followed by 1 week of oral saline or SGE (150 or 300 mg/kg/day). (A) Grip strength measurements. *n* = 6 per group. (B) Representative micro‐CT images of hindlimb skeletal muscle. (C) Representative H&E‐stained sections of the TA muscle (magnification, × 200) and quantification of CSA in the TA muscle fibers. Data are shown as mean ± SEM. *n* = 3 per group. Bars sharing the same letter do not differ significantly (*p* < 0.05) based on one‐way ANOVA with Tukey's post hoc test.

We observed that SGE administration substantially alleviated the immobilization‐induced loss of muscle mass, which can enhance muscle function by providing greater strength, endurance, and overall physical performance. Therefore, we performed a grip strength test to further examine the effect of SGE on muscle function. The IM group showed a significant decrease in both forelimb and hindlimb grip strength, whereas SGE increased grip strength by over 24% relative to the IM group (Cohen's d; −3.70, −5.22 for IM vs. IM + SGE150 or IM + SGE300; Figure 3A). As muscle function closely correlates with density, size, and fiber CSA, we utilized micro‐CT to measure the volume of right hindlimb muscles and performed histological analysis via H&E staining. Compared with the CON group, the IM group exhibited a 22.6% reduction in muscle volume; however, administration of SGE in the IM + SGE150 and IM + SGE300 groups restored muscle volume in a dose‐dependent manner (Figure 3B). Similarly, TA muscle CSA was significantly reduced in the IM group relative to the CON group, but was elevated by 18.7% and 28.2% in the IM + SGE150 and IM + SGE300 groups, respectively (Cohen's d; −3.64, −5.02 for IM vs. IM + SGE150 or IM + SGE300; Figure 3C).

### 3.3 SGE Suppressed Protein Degradation Markers in the TA Muscle Under Immobilization‐Induced Muscle Atrophy

**FIGURE 4:**
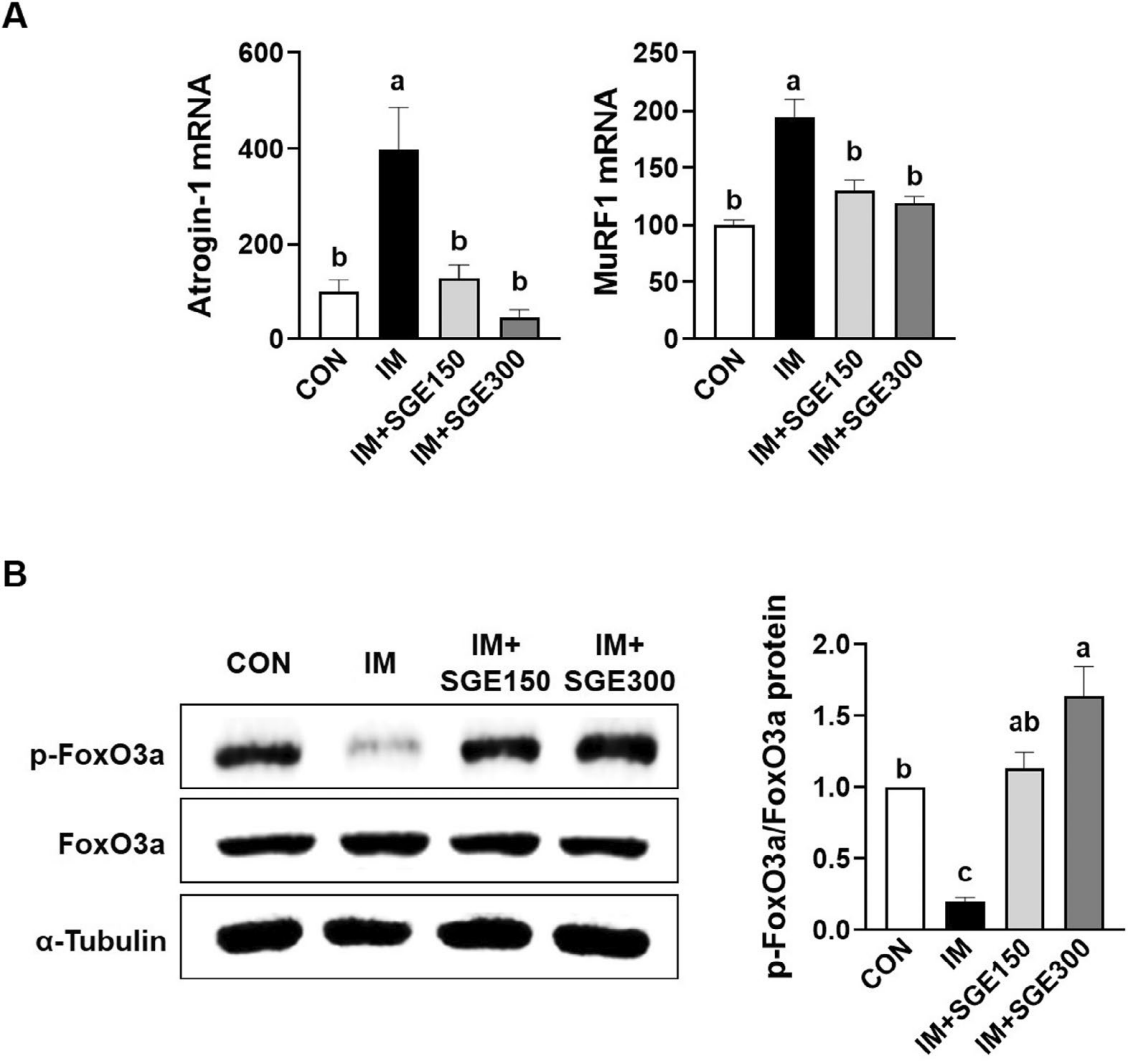
SGE mediated inhibition of protein degradation markers in the TA muscle of mice with immobilization‐induced muscle atrophy. Mice underwent 1 week of hindlimb immobilization to induce skeletal muscle atrophy, followed by 1 week of oral saline or SGE (150 or 300 mg/kg/day). (A) RT‐PCR analysis of MuRF1 and Atrogin‐1 mRNA expression in the TA muscle. (B) Western blot analysis of total and phosphorylated FoxO3a protein levels in the TA muscle. *β*‐Actin and α‐tubulin served as loading controls. Data are shown as mean ± SEM. *n* = 3 per group. Bars sharing the same letter do not differ significantly (*p* < 0.05) based on one‐way ANOVA.

During immobilization‐induced muscle atrophy, muscle‐specific E3 ubiquitin ligases, including muscle ring finger 1 (MuRF1) and muscle atrophy F‐box (Atrogin‐1), are tightly regulated by the transcription factor FoxO3a and become highly expressed (Jackman and Kandarian 2004; Kandarian and Jackman 2006; Kim, Kim, and Hwang 2020). Thus, we next assessed whether SGE can inhibit the elevated protein degradation‐related marker expression in immobilized TA muscles. MuRF1 and Atrogin‐1 mRNA levels were significantly elevated in the IM group but were substantially reduced by SGE administration to near CON levels (Figure 4A). Phosphorylated FoxO3a expression was notably lower in the IM group compared with the CON group, yet significantly recovered following SGE administration (Figure 4B).

### 3.4 SGE Elevated Protein Synthesis Markers in the TA Muscle During Immobilization‐Induced Muscle Atrophy

**FIGURE 5:**
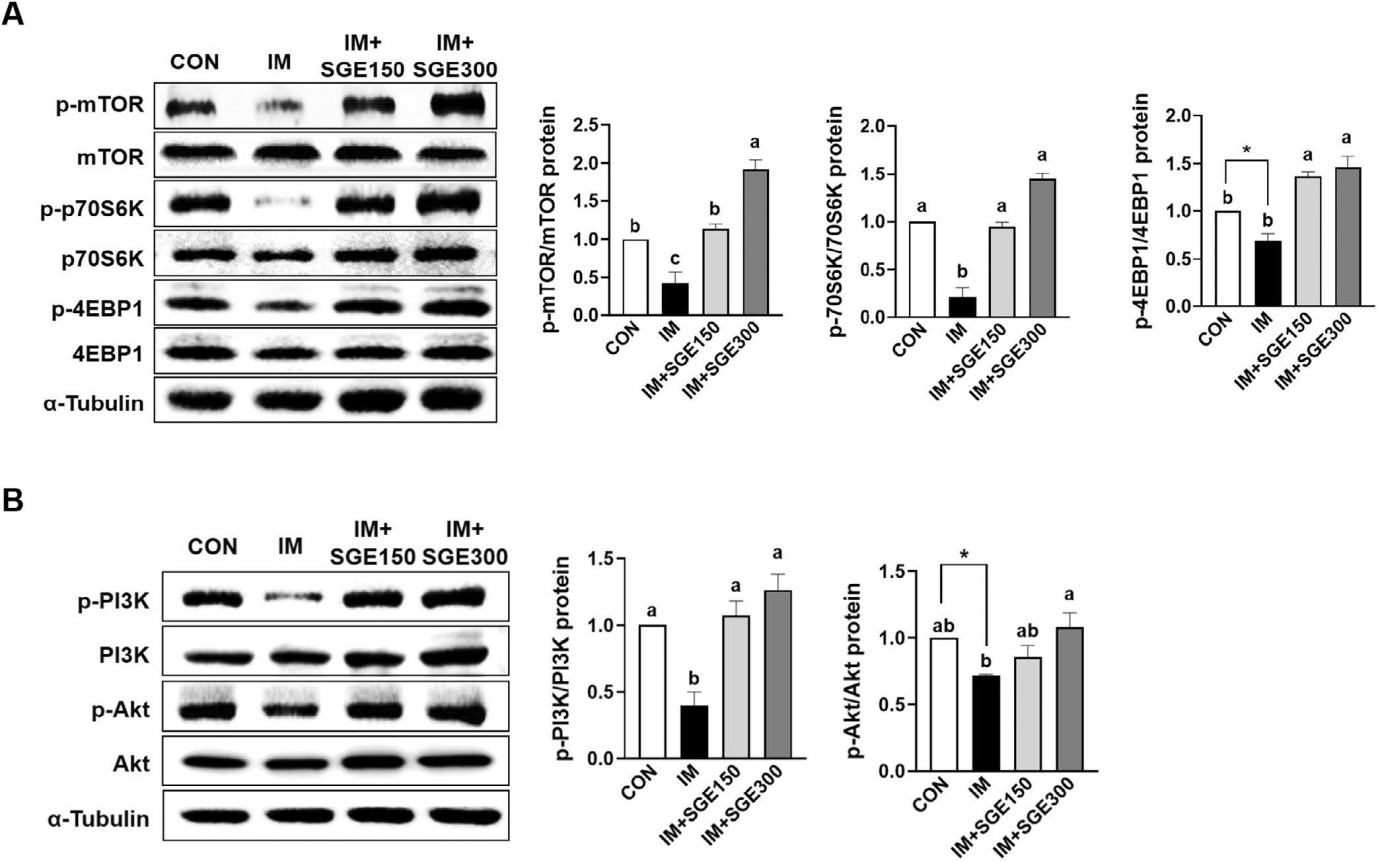
SGE induced enhancement of protein synthesis pathway markers in the TA muscle of mice with immobilization‐induced muscle atrophy. Mice underwent 1 week of hindlimb immobilization to induce skeletal muscle atrophy, followed by 1 week of oral saline or SGE (150 or 300 mg/kg/day). (A) Western blot analysis of total and phosphorylated mTOR, p70S6K, and 4EBP1 in the TA muscle. (B) Western blot analysis of total and phosphorylated Akt and PI3K in the TA muscle. α‐Tubulin was served as loading controls. Data are shown as mean ± SEM. *n* = 3 per group. Bars sharing the same letter do not differ significantly (*p* < 0.05) based on one‐way ANOVA with Tukey's post hoc test or an unpaired *t*‐test.

The mTOR/p70S6K/4EBP1 and PI3K/Akt signaling pathways are pivotal in governing muscle protein synthesis, a process that becomes impaired under immobilization‐induced muscle atrophy (Zhang et al. 2007). Therefore, we evaluated these pathways in the TA muscle of immobilized mice. In comparison with the CON group, the IM group exhibited decreased phosphorylation levels of mTOR, accompanied by diminished phosphorylation of p70S6K and 4EBP1. However, SGE administration completely restored phosphorylated mTOR, p70S6K, and 4EBP1 in the IM + SGE150 and IM + SGE300 groups (Figure 5A). Also, the protein expression levels of PI3K and Akt phosphorylation were significantly suppressed in the IM group compared to the CON group, but were significantly rescued by SGE treatment in the IM + SGE150 and IM + SGE300 groups (Figure 5B).

### 3.5 Kirenol Alleviated TNF‐α‐Triggered Muscle Atrophy in L6 Myotubes

**FIGURE 6:**
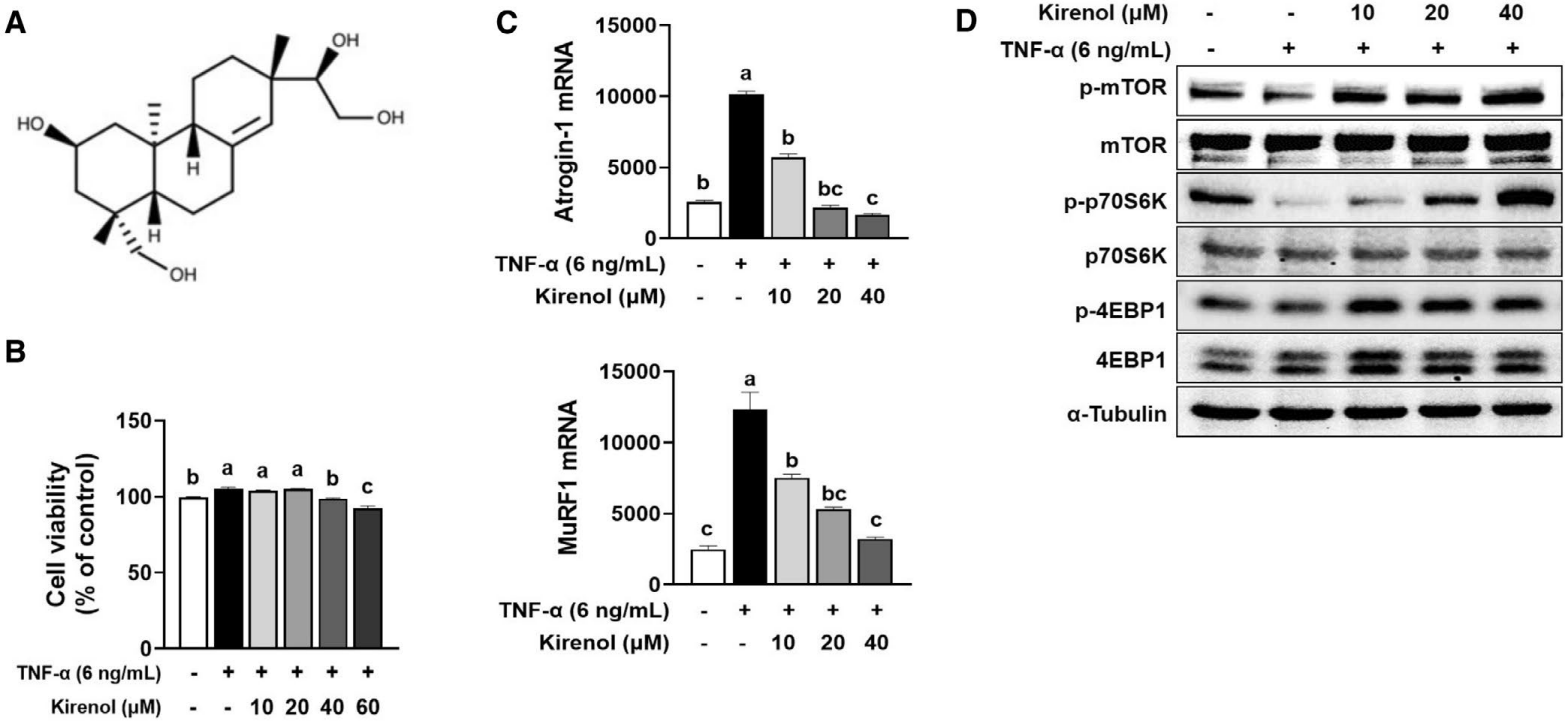
Alleviation of TNF‐α‐induced muscle atrophy by kirenol in L6 myotubes. L6 myotubes were treated with TNF‐α (6 ng/mL) and kirenol (10, 20, and 40 μM) for 24 h. (A) Chemical structure of kirenol. (B) Cell viability. (C) RT‐PCR analysis of MuRF1 and Atrogin‐1 mRNA expression. (D) Western blot analysis of total and phosphorylated mTOR, p70S6K, and 4EBP1. *β*‐Actin and α‐tubulin were employed as loading controls. Data are shown as mean ± SEM. Bars sharing the same letter do not differ significantly (*p* < 0.05) based on one‐way ANOVA with Tukey's post hoc test.

Having observed that SGE effectively countered muscle atrophy caused by immobilization in mice, we further explored whether kirenol (Figure 6A), a main active diterpenoid component in SGE, can inhibit TNF‐α‐induced muscle atrophy in L6 myotubes. When kirenol was treated in L6 myotubes at concentrations ranging 0–60 μM, cell viability at 40 μM of kirenol was approximately 98% (Figure 6B). Consequently, we decided to use kirenol concentrations below 40 μM for the following experiments. The expression of MuRF1 and Atrogin‐1 mRNA was significantly increased by TNF‐α, but it decreased in a dose‐dependent manner with kirenol treatment (Figure 6C). Also, TNF‐α noticeably lowered phosphorylation levels of mTOR, p70S6K, and 4EBP1, whereas kirenol treatment largely restored these phosphorylated proteins (Figure 6D).

## 4 Discussion

During immobilization‐induced muscle atrophy, inflammation and oxidative stress exacerbate muscle loss by disrupting the equilibrium between protein synthesis and degradation (Y. Ji et al. 2022; Lee et al. 2021). Natural products with anti‐inflammatory and antioxidant properties offer promising potential for preventing and treating muscle atrophy. *Siegesbeckia* Herba and its key bioactive constituent, kirenol, have demonstrated potent anti‐inflammatory and antioxidant effects through inhibition of nuclear factor‐κB (NF‐κB) and mitogen‐activated protein kinases (MAPKs), while activating the nuclear factor erythroid 2‐related factor 2 (Nrf2) via the PI3K/Akt signaling pathway (Linghu et al. 2020; Nasir et al. 2022). This observation implies that *Siegesbeckia* Herba and kirenol hold potential for the mitigation of muscle atrophy. However, despite these known properties, further exploration of their effects remains necessary. In this study, we demonstrated that SGE enriched with kirenol alleviates immobilization‐induced skeletal muscle atrophy in C57BL/6J mice by modulating the Akt/mTOR/FoxO3a signaling pathways. Importantly, we also provided the first evidence that kirenol, the diterpenoid of SGE, counters TNF‐α‐induced muscle atrophy in L6 myotubes.

Immobilizing skeletal muscle triggers two primary processes: A suppression of protein synthesis and an upregulation of protein degradation (Ji and Yeo 2019). Protein synthesis is decreased by inhibiting the insulin‐like growth factor 1 (IGF‐1)–Akt–mTOR pathway, while protein degradation is increased by activating the ubiquitin–protein degradation pathway, which is mainly regulated by FoxO subfamily transcription factors (Jackman and Kandarian 2004; Ji and Yeo 2019). Impairment of the equilibrium between protein synthesis and degradation, under conditions where proteolytic activity surpasses anabolic processes, constitutes a principal mechanism underlying the development of muscle atrophy. Akt‐mediated signaling stimulates anabolic processes via the mTOR/p70S6K/4EBP1 axis and concomitantly suppresses proteolysis through FoxO3a phosphorylation, which blocks nuclear translocation and subsequent transactivation of atrogenes encoding ubiquitin ligases, including MuRF1 and Atrogin‐1 (Bodine and Baehr 2014; Sandri et al. 2004). In this study, we observed that SGE promoted the PI3K/Akt/mTOR pathway, increasing the phosphorylation of key downstream substrates p70S6K and 4EBP1 in the TA muscle, which enhances muscle protein synthesis. Continually, SGE reduced the gene expression of MuRF1 and Atrogin‐1 by suppressing FoxO3a phosphorylation, ultimately preventing protein degradation in the TA muscle. Increased protein synthesis coupled with reduced protein degradation promotes the accumulation of total muscle protein and organelles, thereby precipitating greater muscle mass and hypertrophy (Vainshtein and Sandri 2020). Importantly, multiple lines of evidence indicate that kirenol exerts both anti‐inflammatory and antioxidant effects by activating the PI3K/Akt signaling pathway (Nasir et al. 2022). This pathway plays a pivotal role in regulating skeletal muscle mass by promoting protein synthesis and inhibiting protein degradation. In our study, we observed that kirenol enhanced mTOR‐mediated protein synthesis and concurrently suppressed FoxO3a‐driven transcription of MuRF1 and Atrogin‐1, effects that may be mediated by the PI3K/Akt pathway in L6 myotubes. These dual regulatory effects suggest that the beneficial actions of SGE on muscle mass and function are mechanistically linked to these specific signaling cascades. Therefore, the protective effects of SGE against immobilization‐induced skeletal muscle loss are likely attributed to its ability to enhance protein synthesis and decrease protein degradation through the Akt/mTOR/FoxO3a signaling pathway, ultimately increasing muscle mass and volume in mice. However, further research with prolonged treatment and follow‐up is needed to determine whether the beneficial effects of SGE observed in short‐term immobilization are sustained during long‐term disuse conditions.

In this study, SGE administration markedly mitigated the detrimental effects of immobilization, including reductions in TA muscle fiber CSA, decreased muscle weights across four muscle types, and reduced total muscle volume. The decrease in muscle fiber CSA is a well‐established indicator of immobilization‐induced muscle atrophy, reflecting a disruption in the delicate balance between protein synthesis and degradation crucial for maintaining muscle mass (Jackman and Kandarian 2004; Kandarian and Jackman 2006). The protective effect of SGE against muscle atrophy stems from its ability to modulate this balance, primarily through the regulation of the Akt/mTOR/FoxO3a signaling pathways. In particular, kirenol, an active compound of SGE, attenuated TNF‐α‐induced muscle atrophy in L6 myotubes by regulating these same pathways. This observation provides a mechanistic link between kirenol effects at the cellular level and its ability of SGE to maintain muscle mass during immobilization in mice. The preservation of muscle mass and CSA by SGE is particularly important, as muscle mass plays a crucial role in maintaining function and strength. This was evidenced by the substantial alleviation of grip strength loss in mice subjected to immobilization‐induced muscle atrophy following SGE administration. It has been shown that reduced muscle fiber CSA is directly linked to decreased force generation capacity (Kristiansen et al. 2014). The preservation of muscle CSA through SGE administration likely contributes to maintaining muscle function during periods of immobilization. Thus, these findings demonstrate that SGE effectively protects skeletal muscle physiological functions against immobilization stress. This suggests that preserving muscle strength and promoting muscle mass is primarily accomplished through regulation of the Akt/mTOR/FoxO3a signaling pathway. Recent studies have shown that mitochondrial dysfunction, impaired autophagy, and fibrosis are important contributors to muscle wasting (Egerman and Glass 2014; Mahdy 2019; Romanello and Sandri 2016). Although our study focused on the Akt/mTOR/FoxO3a pathway, future research should examine whether SGE and kirenol also influence these pathways, which could provide a more comprehensive understanding of their therapeutic effects in skeletal muscle atrophy.

Immobilization‐induced skeletal muscle atrophy is driven by a combination of chronic inflammation and oxidative stress (Xu et al. 2022). Pro‐inflammatory cytokines are key factors that promote the breakdown of muscle proteins (Sishi and Engelbrecht 2011; Wang et al. 2014). The activation of NF‐κB triggers an increase in the expression of muscle‐specific E3 ubiquitin ligases, which play a crucial role in facilitating protein breakdown within skeletal muscle tissue (Xu et al. 2022). In this study, we found that kirenol attenuated TNFα‐induced muscle atrophy in L6 myotubes by stimulating muscle protein synthesis and inhibiting protein degradation through the Akt/mTOR/FoxO3a signaling pathway. This dual action highlights the ability of kirenol to restore the balance between anabolic and catabolic processes in skeletal muscle. Also, the preventive effect of kirenol on muscle atrophy in L6 myotubes is partly attributed to a reducing proinflammatory cytokines in TNF‐α‐treated L6 myotubes. Furthermore, SGE has been shown to suppress pro‐inflammatory cytokine expression by inhibiting NF‐κB and MAPK signaling pathways, while kirenol enhances antioxidant defenses by activating Nrf2 via PI3K/Akt signaling, reducing intracellular ROS levels (Linghu et al. 2020; Nasir et al. 2022). These protective actions of SGE and kirenol against inflammation and oxidative stress are likely to contribute to their anti‐muscle atrophy effects, which is consistent with previous research showing that natural compounds targeting NF‐κB and oxidative stress pathways can prevent muscle wasting (Nikawa et al. 2021). Hydrogen sulfide donors have been shown to alleviate immobilization‐induced muscle atrophy by reducing inflammation, oxidative stress, and fibrosis (Xu et al. 2022). The ability of SGE to inhibit NF‐κB and MAPK signaling pathways, coupled with the antioxidant properties of kirenol, offers a multi‐faceted approach to combating muscle atrophy. Thus, these results indicate that the anti‐inflammatory and antioxidant properties of SGE and kirenol play a pivotal role in their preventive effects on skeletal muscle atrophy. By targeting key molecular pathways involved in inflammation (NF‐κB) and oxidative stress (Nrf2), SGE and kirenol offer a promising therapeutic approach for managing immobilization‐induced muscle wasting. However, further research is needed to ascertain whether SGE and kirenol exert their effects against muscle atrophy by modulating the NF‐κB and Nrf2 pathways under conditions of immobilization and in TNFα‐induced L6 myotubes.

## 5 Conclusion

In conclusion, this study reveals the novel protective effects of SGE containing kirenol against skeletal muscle atrophy. In mice subjected to immobilization, SGE mitigated the decline in muscle strength, mass, volume, and muscle fiber CSA by inhibiting muscle‐specific E3 ubiquitin ligases and promoting protein synthesis markers through modulation of the Akt/mTOR/FoxO3a pathway. Similarly, kirenol attenuated TNFα‐induced atrophy in L6 myotubes through the same signaling cascade, balancing protein synthesis and degradation. These findings suggest that SGE containing kirenol holds potential for preventing muscle atrophy and may serve as a basis for future development of functional interventions aimed at supporting muscle health.

## Author Contributions

**Sungmin Han:** formal analysis (equal), investigation (equal), methodology (equal), software (equal), visualization (equal), writing – original draft (equal), writing – review and editing (equal). **Jae‐Kwan Hwang:** conceptualization (equal), project administration (equal), resources (equal), supervision (equal), writing – original draft (equal), writing – review and editing (equal). **Mi‐Bo Kim:** conceptualization (equal), data curation (equal), formal analysis (equal), investigation (equal), methodology (equal), software (equal), supervision (equal), visualization (equal), writing – original draft (equal), writing – review and editing (equal).

## Conflicts of Interest

The authors declare no conflicts of interest.

## Supporting information


**Figure S1.** Liver and spleen weight. Mice underwent 1 week of hindlimb immobilization to induce skeletal muscle atrophy, followed by 1 week of oral saline or SGE (150 or 300 mg/kg/day). (A) Liver weight. (B) Spleen weight. Data are shown as mean ± SEM (*n* = 8 per each group). Bars sharing the same letter do not differ significantly (*p* < 0.05) based on one‐way ANOVA with Tukey's post hoc test. *p* values for the unpaired *t*‐test are indicated.

## Data Availability

This study is available from the corresponding author upon reasonable request.
