# Supplementary material for: Siegesbeckia Glabrescens Extract Ameliorates Immobilization‐Induced Muscle Atrophy by Regulating the Akt/mTOR/FoxO3a Signaling Pathways in Mice
Source: Food Sci Nutr. 2025 Sep 26;13(10):e71005. doi: 10.1002/fsn3.71005 (PMC12464453; doi:10.1002/fsn3.71005)
Supplement: Supplementary file 1 — Figure S1. Liver and spleen weight. Mice underwent 1 week of hindlimb immobilization to induce skeletal muscle atrophy, followed by 1 week of oral saline or SGE (150 or 300 mg/kg/day). (A) Liver weight. (B) Spleen weight. Data are shown as mean ± SEM (n = 8 per each group). Bars sharing the same letter do not differ significantly (p < 0.05) based on one‐way ANOVA with Tukey's post hoc test. p values for the unpaired t‐test are indicated. [file FSN3-13-e71005-s001.docx]

**A B**

**Figure S1** Liver and spleen weight. Mice underwent 1 week of hindlimb immobilization to induce skeletal muscle atrophy, followed by 1 week of oral saline or SGE (150 or 300 mg/kg/day). (A) Liver weight. (B) Spleen weight. Data are shown as mean ± SEM (*n* = 8 per each group). Bars sharing the same letter do not differ significantly (*P* < 0.05) based on one-way ANOVA with Tukey’s post hoc test. *P*-values for the unpaired *t*-test are indicated.
